# Prevalence of primary bacterial co-infections among patients with COVID-19 in Brunei Darussalam

**DOI:** 10.5365/wpsar.2021.12.3.856

**Published:** 2021-09-08

**Authors:** Aieman Bashir, Muhammad Syafiq Abdullah, Natalie Raimiza Momin, Pui Lin Chong, Rosmonaliza Asli, Babu Mani Ivan, Vui Heng Chong

**Affiliations:** a Department of Medicine, Pengiran Muda Mahkota Pengiran Muda Haji Al-Muhtadee Billah Hospital, Tutong, Brunei Darussalam.; b Department of Medicine, Raja Isteri Pengiran Anak Saleha Hospital, Bandar Seri Begawan, Brunei Darussalam.

## Abstract

**Objective:**

Bacterial co-infections in cases of coronavirus disease 2019 (COVID-19) can lead to less favourable outcomes. The aim of this study was to determine the prevalence of primary bacterial co-infections among patients with COVID-19 in Brunei Darussalam.

**Methods:**

Seventy-one of 180 patients admitted to the National Isolation Centre between 9 March 2020 and 4 February 2021 were screened for primary bacterial co-infection (infection occurring £48 hour from admission). We compared patients with a primary bacterial co-infection to those without.

**Results:**

Of the 71 screened patients, 8 (11.2%) had a primary bacterial co-infection (sputum 37.5% [6/16], blood 2.8% [1/36], urine 1.7% [1/60]), for a period prevalence rate of 4.4% (respiratory tract infection 3.3% [6/180], bloodstream 0.6% [1/180], urine 0.6% [1/180]) among all COVID-19 patients. Older age, presence of comorbidity, symptoms at admission (fever, dyspnoea, nausea/vomiting), abnormal chest X-ray (CXR) and more severe COVID-19 (*P* < 0.05) were associated with primary bacterial co-infection. Primary bacterial co-infection was also associated with development of secondary infection and death (all *P* < 0.05). Only one patient with primary bacterial co-infection died (methicillin-sensitive *Staphylococcus aureus* septicaemia and multiorgan failure).

**Conclusion:**

Our study showed that primary bacterial co-infection affected 4.4% of patients with COVID-19 in Brunei Darussalam. Older age, presence of comorbidity, symptoms and abnormal CXR at admission and more severe disease were associated with a primary bacterial co-infection. Lower respiratory tract infection was the most common co-infection.

Coronavirus disease 2019 (COVID-19), caused by severe acute respiratory syndrome coronavirus 2 (SARS-CoV-2), is associated with significant morbidity and mortality, especially among susceptible populations such as the elderly and those with comorbidities. (*1*) Co-infections have been reported in COVID-19 patients and can lead to less favourable outcomes. (*2*-*5*) To date, most studies have reported overall co-infection rates and have not distinguished between primary and later co-infections. Primary or early co-infection is defined as any infection occurring simultaneously or detected soon after admission, usually within 48 or 72 hour. Any co-infection occurring after this period is regarded as secondary co-infection.

Brunei Darussalam reported its first COVID-19 case on 9 March 2020, and, as of 4 February 2021, had recorded 180 cases. The country remains at WHO level 2 of transmission, (*6*) with sporadic imported cases and no local infection since 6 May 2020. All patients diagnosed with COVID-19, regardless of symptoms, are admitted to the National Isolation Centre (NIC) for isolation and treatment. We report our nationwide experience in screening for primary bacterial co-infection among patients treated for COVID-19 in Brunei Darussalam.

## Methods

In this descriptive study, data were retrospectively retrieved from a prospectively maintained database. This Excel® database was created at the start of the COVID-19 outbreak to monitor patients and for situational management and analysis. The information collected includes demographic data, comorbidities, symptoms at admission and during hospitalization, investigations, treatment and outcomes.

### COVID-19 management and categorization

In Brunei Darussalam, all patients who test positive for SARS-CoV-2 by reverse transcription polymerase chain reaction (RT–PCR) are admitted to the NIC (designated hospital for COVID-19 for the whole country) for isolation and treatment.

Patients were categorized as symptomatic if they had symptoms at admission, asymptomatic if they had no symptoms during their illness and pre-symptomatic if they were asymptomatic at admission but developed symptoms during hospitalization. Management was based on the treatment protocol described in our previous publication. (*7*)

Patients were categorized into three levels of disease severity: mild (asymptomatic or mild disease, i.e. with none of the features listed under the moderate and severe categories), moderate (fever with respiratory tract symptoms and pneumonia on imaging) and severe (respiratory rate > 30/min, oxygen saturation £ 93% at rest, *Pa*o_2_/FiO2 £ 300 mm Hg and progression of lung lesions > 50% within 24–48 hour). Patients categorized as having mild COVID-19 were managed in the main isolation wards, and those with moderate or severe COVID-19 were managed in the high-dependency or intensive-care setting.

### Screening for primary bacterial co-infection

Primary bacterial co-infection was defined as infection diagnosed within 48 hour of admission. Screening for primary bacterial co-infection was carried out according to our management protocol. (*7*) At admission, a detailed history and clinical examinations were carried out for all patients, and all had routine investigations (blood and chest X-ray [CXR]). Patients with fever, rigor, cough, shortness of breath, dysuria, diarrhoea, abnormal investigational findings (leukocytosis, elevated inflammatory markers [C-reactive protein], elevated procalcitonin) and abnormal CXR were screened for primary co-infection by collection of sputum, urine and stool for Gram staining, microscopy and culture, and blood for culture.

### Management of co-infections

As our management protocol included a 5-day course of oseltamivir to cover influenza A and B, we did not screen for influenza viruses. We also did not screen for other common respiratory viral pathogens, as no specific treatment is available. After investigations for primary bacterial co-infection, empiric antimicrobials were administered when indicated and later adjusted according to culture sensitivity patterns for those who screened positive. Patients who developed or continued to have symptoms 48 hour after admission were screened for secondary co-infections, defined as infection occurring more than 48 hour after admission. Clinical management was guided by the investigational results.

### Data analyses

Data were anonymized and extracted from our Excel® database for analysis with IBM SPSS version 23.0. In our management protocol, only patients who had symptoms or abnormal findings of investigations at admission were screened for primary bacterial co-infections. Patients who were not screened were included in the group with no primary bacterial co-infections for analyses. We assessed factors associated with positivity for primary bacterial co-infection (demographic characteristics, symptoms, investigations, disease severity) and outcomes (screening and positivity for secondary co-infection, death) using the Mann–Whitney test for continuous variables and the Fisher exact test for categorical variables. A *P*-value of < 0.05 was considered statistically significant.

## Results

As of 4 February 2021, 180 patients (mean age, 35.3 ± 16.7 years; males, 62.2%) had been admitted to the NIC with COVID-19. Among them, 28.9% (*n* = 52) had comorbidities, including hypertension (13.3%), dyslipidaemia (13.9%), diabetes mellitus (5.6%), pulmonary disease (4.4%) and cardiac disease (3.3%). Overall, 56.1% (*n* = 101) were symptomatic, 6.7% (*n* = 12) were pre-symptomatic and 37.2% (*n* = 67) were asymptomatic. Common symptoms included cough (33.9%), fever (23.3%) and rhinorrhoea (20.0%). An abnormal CXR at admission was noted in 15.3%, and the cycle threshold value was 27.2 ± 6.3. The majority of cases were categorized as mild, with 11.7% categorized as moderate and 2.7% as severe (**Table 1**).

**Table 1:** Characteristics and outcomes of patients

Variable	All patients (*n* = 180) *n*(%)	Positive for primary co-infection (*n* = 8)	Negative for primary co-infection (*n* = 172)	*P*
Demography and characteristics				
Mean age (years)	35.3 ± 16.7	50.6 ± 12.6	34.6 ± 16.5	0.007
Median (range (years))	33 (0.58–72)	53 (29–64)	32 (0.58–72)	
Gender				
Male	112 (62.2)	6 (75.0)	106 (61.6)	0.446
Female	68 (37.8)	2 (25.0	66 (38.4)	-
Comorbidity (yes)	52 (28.9)	5 (62.5)	47 (27.3)	0.032
Hypertension	24 (13.3)	4 (50.0)	20 (11.6)	0.002
Diabetes	10 (5.6)	2 (25.0)	8 (4.7)	0.014
Dyslipidaemia	25 (13.9)	5 (62.5)	20 (11.6)	< 0.001
Cardiac disease	6 (4.3)	1 (12.5)	5 (2.9)	0.14
Pulmonary disease	8 (4.4)	1 (12.5)	7 (4.1)	0.259
Symptoms at admission (yes)	101 (56.1)	6 (75.0)	95 (55.2)	0.271
Fever	42 (23.3)	5 (62.5)	37 (21.5)	0.007
Cough	61 (33.9)	4 (50.0)	57 (33.1)	0.325
Rhinorrhoea	36 (20.0)	2 (25.0)	34 (19.8)	0.718
Diarrhoea	9 (5.0)	1 (12.5)	8 (4.7)	0.319
Anosmia	2 (1.1)	0 (0.0)	2 (1.2)	0.759
Myalgia	16 (8.9)	1 (12.5)	15 (8.7)	0.714
Sore throat	12 (6.7)	0 (0.0)	12 (7.0)	0.439
Headache	13 (7.2)	0 (0.0)	13 (7.6)	0.419
Nausea/vomiting	3 (1.7)	1 (12.5)	2 (1.2)	0.014
Dyspnoea	4 (2.1)	3 (37.5)	1 (0.6)	< 0.001
Admission investigations				
Chest X-ray*				
Abnormal	26 (15.3)	4 (50.0)	22 (13.6)	0.005
Investigations	-	-	-	-
Cycle threshold	27.2 ± 6.3	25.3 ± 6.8	27.2 ± 6.3	0.336
Albumin	39.4 ± 3.9	38.3 ± 2.9	39.4 ± 3.97	0.314
Neutrophil	3.8 ± 2.2	4.10 ± 2.93	3.82 ± 2.16	0.94
Lymphocyte	2.0 ± 0.8	1.61 ± 0.66	1.97 ± 0.77	0.294
Neutrophil-lymphocyte ratio	2.39 ± 2.39	3.17 ± 2.65	2.35 ± 2.38	0.429
Outcomes				
Disease category	-	-	-	-
Mild	154 (85.6)	4 (50.0)	150 (87.2)	0.012 for trend
Moderate	21 (11.7)	3 (37.5)	18 (10.5)	
Severe/critical	5 (2.7)	1(12.5)	4 (2.3)	
Screened for secondary co-infection	20 (11.1)	3 (37.5)	17 (9.9)	0.015
Developed secondary co-infection	10 (5.6)	2 (25.0)	8 (4.7)	0.014
Death (mortality rate)	3 (1.7)	1 (12.5)	2 (1.2)	0.014

*Ten patients did not undergo chest X-ray.

Primary bacterial co-infection was screened in 71 (39.4%) patients. Of these, 80.3% (57 patients) were screened within 24 hour and 19.7% (14 patients) 24–48 hour after admission. Of the 71 patients screened, 8 (11.2%) had a primary bacterial co-infection, usually in the lower respiratory tract, for a period prevalence of 4.4% among all COVID-19 patients. Most of the positive samples were sputum (6/16 patients [37.5%]), followed by blood (1/36 patients [2.8%]) and urine (1/60 patients [1.7%]). Stool assessment showed no bacteria. The bacteria isolated consisted of *Klebsiella* species (2 patients), *Streptococcus* spp. (2 patients), methicillin-resistant *Staphylococcus* species (2 patients), *Enterobacter* species (2 patients), *Rothia mucilaginosa* (1 patient) and *Haemophilus influenzae* (1 patient). Two organisms were isolated from two patients (cases 3 and 4 in **Table 2**).

**Table 2:** Details of screening and result for primary bacterial co-infections

Sample	*n*(%) (*n* = 180)	Positivity rate *n*(%)	Case no.	Organism/s	Outcomes
Blood	36 (20.0)	1 (2.8)	1	Methicillin-sensitive *Staphylococcus aureus*	Died of septicaemia/ARDS
Urine	60 (33.3)	1 (1.7)	2	*Streptococcus* group B	Treated and alive
Sputum	16 (8.9)	6 (37.5)	3	*Rothia mucilaginosa*/*Enterobacter gergoviae*	All treated and alive
			4	*Klebsiella pneumoniae*/MRSA	
			5	*Haemophilus influenzae*	
			6	*Streptococcus pneumoniae*	
			7	*Klebsiella pneumoniae*	
			8	*Enterobacter aerogenes*	
Stool	2 (1.1)	0 (0.0)	-	Negative	–

ARDS: acute respiratory distress syndrome; MRSA: methicillin-resistant *Staphylococcus aureus.*

One patient (case 4) with methicillin-resistant *Staphylococcus aureus* (MRSA) isolated from sputum had a history of clinic visits, and screening showed colonization only in the nose. The patient (case 2) with group B *Streptococcus* isolated in urine reported no urinary symptoms but had fever, myalgia and nausea at admission. He was treated for urinary infection with a course of co-amoxiclav. Blood and sputum cultures from this patient were negative.

Patients with primary bacterial co-infection tended to be older, had comorbidities (especially hypertension, diabetes mellitus and dyslipidaemia), reported symptoms at admission (fever, nausea/vomiting, dyspnoea), had abnormal CXR and were categorized as having moderate or severe disease (**Table 1**). These patients were also more likely than those without primary bacterial co-infection to be screened for (37.5% versus 9.9%, *P* = 0.015) and develop secondary co-infection (25% versus 4.7%, *P* = 0.014) and to die (12.5% versus 0.6%, *P* = 0.014).

One of the patients with primary bacterial co-infection died 16 days after admission due to MRSA septicaemia. This patient presented late (5 days after the onset of dyspnoea), was symptomatic at admission and deteriorated rapidly. The CXR was consistent with pneumonia. Sputum Gram staining was positive for Gram-positive cocci, but no organism was isolated. All the other patients with primary bacterial co-infection recovered.

## Discussion

We assessed the period prevalence of primary bacterial co-infection, factors and outcomes among patients with COVID-19 in Brunei Darussalam. The period prevalence was 4.4%, consistent with the results of other published studies. In one study in Spain, 37 primary co-infections were reported in 31 (of 989) patients admitted with COVID-19, a prevalence of 3.1%; 30 were bacterial co-infections (in 25 patients) and 7 were viral co-infections. Two patients among those with bacterial co-infection were infected with two different organisms. (*8*) A study in the United States of America found that 2.8% of COVID-19 patients had primary respiratory tract co-infection and 1.1% had bacteraemia. The authors screened 80 (14.7%) of 542 patients with COVID-19 for primary bacterial respiratory co-infection, of whom 15 (2.8%) were treated as having true primary respiratory co-infections, defined as any infection identified within 72 hour of admission. (*9*) Although we did not screen patients for viral co-infection, the rate has been reported to be 1.5–3.0%. (*2*, *10*, *11*) Respiratory syncytial virus and influenza A are the two most common, with detection rates of 16.9% and 15.5%, respectively. (*2*)

Most of the co-infections in our study were of the lower respiratory tract. As COVID-19 is mainly a respiratory illness, primary and secondary bacterial co-infections can occur, as in other respiratory viral illnesses. (*2*, *3*, *8*, *9*) Therefore, it is not surprising that we found that the respiratory tract was the most common site of bacterial co-infection and that sputum samples had the highest proportion of positive results.

Overall, a wide spectrum of organisms has been found in COVID-19 co-infections, with some patients having more than one co-infection. In a study in China, 24 types of respiratory pathogen were found in respiratory samples from patients with COVID-19, of whom 94.2% were co-infected with one or more pathogens. Common bacterial pathogens reported included *S. pneumoniae*, *K. pneumoniae* and *H. influenzae*. (*12*) In our study, several organisms were isolated from two patients: *R. mucilaginosa* and *E. gergoviae* (case 3) and MRSA and *K. pneumoniae* (case 4), which are uncommon organisms. The reported spectrum of organisms in nosocomial co-infections among hospitalized COVID-19 patients is similar to those encountered in clinical practice among non-COVID-19 patients. (*13*, *14*)

Although few bacterial and viral co-infections have been identified in COVID-19 patients, vigilance must be maintained, with screening for both primary and secondary co-infections, as they have been associated with less favourable outcomes. (*9*) In our study, COVID-19 patients with primary bacterial co-infection were older, had comorbidities, presented with fever, dyspnoea and nausea/vomiting, had an abnormal CXR at admission and were categorized as having moderate or severe disease. COVID-19 patients with primary bacterial co-infection were also more likely to be screened for and develop secondary infection and were more likely to die. One systematic review and meta-analysis showed that the odds of death were higher for patients with co-infections and superinfection than for those with only SARS-CoV-2 infection (odds ratio = 3.31; 95% confidence interval, 1.82–5.99). (*15*)

Strengths and limitations of our study should be considered when interpreting the findings. The main strength of our study is that it included all COVID-19 cases in the country and may therefore be applicable to countries or regions with similar demographics, health-care infrastructure and management of the COVID-19 pandemic. An important limitation is the small numbers of COVID-19 patients and of primary bacterial co-infections, as small samples make the results liable to inherent errors in statistical analysis. Another limitation is the evolving nature of our management protocol in the earlier phase of the outbreak. Apart from normal symptom screening and investigations, some patients also underwent opportunistic screening of easily obtained samples, such as urine. Furthermore, not all our patients were screened, and patients with mild or asymptomatic primary bacterial co-infection might have been missed. The proportion is, however, likely small. Importantly, our study represents a real-world situation in which screening is conducted mainly when indicated.

In conclusion, we have shown that primary bacterial co-infection, mostly in the respiratory tract, has affected 4.4% of COVID-19 patients in Brunei Darussalam. The rate of primary bacterial co-infection was higher among older patients and those with comorbidities, symptoms at admission (particularly fever, nausea/vomiting and dyspnoea), an abnormal CXR and moderate or severe disease. Primary bacterial co-infection was also associated with secondary co-infection and death. Routine blood and urine screening for bacterial co-infection is not indicated and should be guided by clinical and laboratory indicators; however, primary co-infections should be identified early and treated appropriately to prevent complications.
